# Evaluation of A Baculovirus-Expressed VP2 Subunit Vaccine for the Protection of White-Tailed Deer (*Odocoileus virginianus*) from Epizootic Hemorrhagic Disease

**DOI:** 10.3390/vaccines8010059

**Published:** 2020-01-31

**Authors:** Sun Young Sunwoo, Leela E. Noronha, Igor Morozov, Jessie D. Trujillo, In Joong Kim, Erin E. Schirtzinger, Bonto Faburay, Barbara S. Drolet, Kinga Urbaniak, D. Scott McVey, David A. Meekins, Mitchell V. Palmer, Velmurugan Balaraman, William C. Wilson, Juergen A. Richt

**Affiliations:** 1 Center of Excellence for Emerging and Zoonotic Animal Diseases, and the Department of Diagnostic Medicine/Pathobiology, College of Veterinary Medicine, Kansas State University, Manhattan, KS 66506, USA; sunwoosy@gmail.com (S.Y.S.); imorozov@vet.k-state.edu (I.M.); jdtrujillo@vet.k-state.edu (J.D.T.); lui0125@gmail.com (I.J.K.); bfaburay@vet.k-state.edu (B.F.); kinga.urbaniak@piwet.pulawy.pl (K.U.); dmeekins@vet.k-state.edu (D.A.M.); balarama@vet.k-state.edu (V.B.); 2 United States Department of Agriculture, Agricultural Research Service, Center for Grain and Animal Health Research, Arthropod-Borne Animal Diseases Research Unit, Manhattan, KS 66506, USA; leela.noronha@usda.gov (L.E.N.); eesem@k-state.edu (E.E.S.); barbara.drolet@usda.gov (B.S.D.); scott.mcvey@usda.gov (D.S.M.); 3 United States Department of Agriculture, Agricultural Research Service, National Animal Disease Center, Infectious Bacterial Diseases Research Unit, Ames, IA 50010, USA; mitchell.palmer@usda.gov

**Keywords:** white-tailed deer, cattle, epizootic hemorrhagic disease virus, subunit vaccine

## Abstract

Epizootic hemorrhagic disease virus (EHDV) is an arthropod-transmitted RNA virus and the causative agent of epizootic hemorrhagic disease (EHD) in wild and domestic ruminants. In North America, white-tailed deer (WTD) experience the highest EHD-related morbidity and mortality, although clinical disease is reported in cattle during severe epizootics. No commercially licensed EHDV vaccine is available in North America. The objective of this study was to develop and evaluate a subunit vaccine candidate to control EHD in WTD. Recombinant VP2 (rVP2) outer capsid proteins of EHDV serotypes 2 (EHDV-2) and 6 (EHDV-6) were produced in a baculovirus-expression system. Mice and cattle vaccinated with EHDV-2 or EHDV-6 rVP2 produced homologous virus-neutralizing antibodies. In an immunogenicity/efficacy study, captive-bred WTD received 2 doses of EHDV-2 rVP2 or sham vaccine, then were challenged with wild-type EHDV-2 at 30 d post vaccination. None of the rVP2-vaccinated deer developed clinical disease, no viral RNA was detected in their blood or tissues (liver, lung, spleen, kidney), and no EHDV-induced lesions were observed. Sham-vaccinated deer developed clinical disease with viremia and typical EHD vascular lesions. Here, we demonstrate a rVP2 subunit vaccine that can provide protective immunity from EHDV infection and which may serve as an effective tool in preventing clinical EHD and reducing virus transmission.

## 1. Introduction

Epizootic hemorrhagic disease (EHD) is an arthropod-transmitted viral disease of wild and domestic ruminants. The causative agent of EHD is epizootic hemorrhagic disease virus (EHDV; genus *Orbivirus*, family *Reoviridae*), a non-enveloped dsRNA virus closely related to bluetongue virus (BTV) [1]. The geographic distribution of EHDV is broad and includes Africa, the Americas, Australia, Asia, and the Mediterranean basin [2]. With a few exceptions, this distribution is related to the presence of its arthropod vector, namely hematophagous biting midges of the genus *Culicoides* [3]. The numerous strains of EHDV are categorized into seven serotypes: 1, 2, and 4-8 [4], with serotype 1, 2, and 6 viruses known to circulate in North America [5]. Serotype 2 (EHDV-2) was first isolated in North America in 1962 in Alberta, Canada, and has been the predominant serotype identified in EHD outbreaks in recent years in the United States (U.S.) [6,7,8]. However, surveillance studies indicate that serotype 6 (EHDV-6), which was first detected in the U.S. in 2006, likely has since become established throughout the country [9].

EHD has been documented in multiple cervid and bovid species, but primarily impacts North American white-tailed deer (WTD; *Odocoileus virginianus*) [10]. EHD can occur as peracute, acute, or chronic disease syndromes with considerable variation in presentation between geographic regions. In the southern U.S., disease may be asymptomatic or mild as a result of prior exposure of susceptible animals, whereas in more northern areas where exposure is sporadic, severe cases of EHD with hemorrhagic manifestations and death occur more frequently [11]. However, the patterns of disease are dynamic, and there is evidence that the frequency, severity, and geographic range of EHD has increased in recent decades [8]. In susceptible wildlife populations, outbreak-related mortality rates of up to 20% have been estimated [12]. In North America, EHD epizootics have also had substantial effects on commercial deer farming—a rapidly growing industry that had an estimated $7.9 billion total economic impact on the U.S. economy in 2015 [13]. There is evidence that farmed deer may experience higher EHDV exposures than wild deer living in adjacent areas [14].

Clinical disease is typically less common in EHDV-infected cattle, with some notable exceptions. In Asia, Ibaraki virus (IBAV), a strain of EHDV-2, sporadically causes Ibaraki disease—an acute febrile disease which can resemble bluetongue [15]. Reports of bluetongue-like clinical disease in cattle during recent EHDV outbreaks in North America, the Mediterranean Basin, and Reunion Island have also raised concerns about an apparent increase in pathogenicity of additional virus strains in cattle [5,16,17]. EHDV can produce oral lesions in cattle that can resemble those of transboundary diseases such as foot-and-mouth disease, thus EHD-affected premises can be subject to foreign animal disease investigation and animal movement restrictions.

Despite these impacts of EHDV infection on livestock production systems, no commercial EHDV vaccines are currently licensed for use in North America; only inactivated autogenous vaccines have been available [18]. In Japan, inactivated and live–attenuated (modified live) virus vaccines are available for control of IBAV; however, these vaccines do not permit the differentiation between infected and vaccinated animals (DIVA), which is necessary for some intervention strategies. Application of recombinant subunit protein based-vaccines represents an attractive alternative with the potential to offer DIVA compatibility and increased safety, commonly not associated with live-attenuated and killed vaccines [19]. 

The EHDV genome is comprised of 10 linear gene segments, which encode 7 structural proteins of the outer and inner virus capsids (VP1-7) and 4 nonstructural proteins (NS1-3/3a) [1,20]. Previously, it has been demonstrated that subunit vaccines containing multiple capsid proteins of the orbiviruses BTV and African horse sickness virus (AHSV) can be immunogenic and efficacious *in vivo* (reviewed in [21,22,23]). Additionally, immunization with the outer capsid VP2 protein only, which contains the primary antigenic determinants for host-neutralizing antibodies, has been shown to protect susceptible species from experimental BTV and AHSV challenges [24,25]. To date, EHDV subunit vaccines that have been tested for immunogenicity or efficacy in naturally susceptible species have not been described.

The primary objective of this study was to generate a safe and efficacious subunit vaccine for the protection of WTD against EHD. To this end, recombinant VP2 (rVP2) proteins from EHDV-2 and EHDV-6 were produced using a baculovirus expression system. Purified rVP2 proteins, formulated with adjuvant, were evaluated for immunogenicity in mice and cattle. Also, an immunogenicity and efficacy study in WTD was performed using the EHDV-2 rVP2 vaccine candidate administered twice prior to challenge with a pathogenic EHDV-2 strain. Clinical, immunological, and virological parameters were evaluated for 14 days post challenge (dpc), including postmortem macroscopic and microscopic examination. 

## 2. Materials and Methods 

### 2.1. Ethics Statement

All animal studies were carried out in accordance with guidelines set forth by the Animal Welfare Act, The Guide for the Care and Use of Laboratory Animals, 8th edition and/or The Guide for the Care and Use of Agricultural Animals in Research and Teaching, 3rd edition, as applicable for each species. Approval and oversight was granted by the Kansas State University Institutional Biosafety (IBC) and Animal Care and Use (IACUC) Committees. The experimental work described herein falls under KSU IBC protocol #1108 (approved 04/06/2016), and IACUC protocols #3721 (approved 03/07/2016), #3846 (approved 02/15/2017), and #3999 (approved 11/20/2017). 

### 2.2. Generation of EHDV Recombinant Proteins

**Figure 1:**
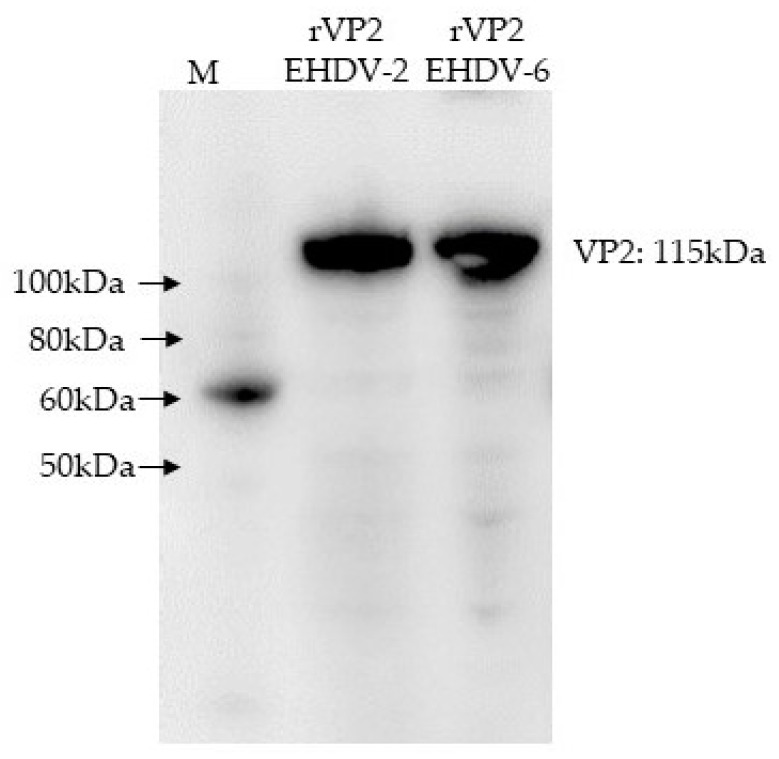
Western blot of baculovirus-expressed epizootic hemorrhagic disease virus (EHDV)-2 rVP2 and EHDV-6 rVP2. Western blot was performed using an anti-histidine tag-HRP antibody. The target size of expressed VP2 proteins is approximately 115kDa. M: protein ladder (SuperSignal™ Molecular Weight Protein Ladder/ThermoFisher).

Total nucleic acid was extracted from EHDV-2 (Alberta 1962, SV-124-Canada) and EHDV-6 (Indiana 2012, 12-38993). Viruses were obtained from the Arthropod-Borne Animal Diseases Research Unit (ABADRU) and Southeastern Cooperative Wildlife Disease Study (SCWDS), and double-stranded RNA was purified by lithium chloride differential precipitation as described previously [26]. Full-length VP2 cDNA of serotype 2 was amplified using the SuperScript III one-step RT-PCR system (ThermoFisher Scientific, Carlsbad, CA), cloned into the pGEM-T vector (Promega, WI), then subcloned into the pENTR/D Topo vector (ThermoFisher). Full-length VP2 cDNA of serotype 6 was amplified using the SuperScript IV reverse transcriptase (ThermoFisher), followed by Q5 high-fidelity DNA polymerase amplification (NEB Inc., Ipswich, MA), and then cloned into the pENTR/D Topo vector. The insertion and orientation of the respective genes in donor plasmids were confirmed by restriction enzyme analysis of the PCR-amplified gene and DNA sequencing (GENEWIZ, South Plainfield, NJ). pENTR-VP2 plasmids were propagated by transforming into One Shot TOP10 chemically competent *E. coli*, following purification using the Qiagen Midiprep kit (Qiagen, Valencia, CA), then incubated with Baculo-Direct linearized baculovirus DNA (ThermoFisher) containing Histidine Tag and LR clonase II to generate the recombinant baculovirus DNA. The recombinant baculovirus DNA was transfected into *Spodoptera frugiperda* 9 (Sf 9) cells using Cellfectin II reagent (ThermoFisher). Plasmid-transfected Sf9 cells were cultured in SF900II medium (ThermoFisher) with 100 μM of ganciclovir to select recombinant baculoviruses, and expression of recombinant proteins was confirmed via Western blot of infected Sf9 cell lysates using an anti-His (C-term)-horseradish peroxidase (HRP) monoclonal antibody (Figure 1) and polyclonal anti-EHDV sera from deer. After confirmation of expression, recombinant proteins with histidine tags were purified via affinity chromatography using Ni-NTA superflow resin (Qiagen) after lysis of cells under native conditions. Cell pellets were resuspended in 50 mM sodium phosphate, 500 mM sodium chloride, 10 mM imidazole, 10% glycerol, and 400 units benzonase nuclease per 1 gram of pellet, at pH 8.0, and followed by twice freezing and thawing and 6 times short (10 sec) sonication. The lysate was centrifuged at 10,000 rpm, at 4 °C for 20 min, and the supernatant was used for Ni-NTA resin purification. Purified proteins were dialyzed against phosphate-buffered saline (PBS; pH 7.4, 150mM NaCl, 4mM EDTA, 10% glycerol). Purification of EHDV proteins was confirmed with Coomassie blue staining of SDS-PAGE gels and EHDV- or HisTag-specific antibodies in Western blots. Protein concentrations were determined using the bicinchoninic acid (BCA) or Braford assay (ThermoFisher) at an absorbance of 562 nm, using bovine serum albumin (Sigma-Aldrich) as the protein standard. Protein aliquots were stored at −80 °C until use.

### 2.3. Animals and Experimental Design

#### 2.3.1. Mice

Immunogenicity of the EHDV rVP2 proteins was tested in two studies utilizing mice. For both experiments, female CD1-ISG mice aged 6 weeks from Charles River Laboratories were randomized into groups of five. Study 1: Mice were immunized subcutaneously (SC) on day 0 with 20 μg of purified EHDV-2 rVP2 formulated with 25% volume of Montanide ISA25 adjuvant (Seppic, France) in a total volume of 200 μL. Sham-vaccinated controls received placebo (1x PBS in adjuvant) SC. A third group was immunized SC with 10^5^ TCID_50_ formaldehyde-inactivated EHDV-2 with adjuvant. Day 21 after first vaccination, identical booster SC vaccinations were administered. Saphenous blood was collected on 0, 21, and 35 days post vaccination (dpv), and mice were euthanized on 35 dpv. Study 2: Mice were immunized SC on day 0 with 20 μg of purified EHDV-6 rVP2 formulated with 25% volume of Montanide ISA25 adjuvant (Seppic, France) in a total volume of 200 μL. Sham vaccinated mice received placebo (1x PBS in adjuvant) SC. A third group was immunized SC with 10^5^ TCID_50_ formaldehyde-inactivated EHDV-6 with adjuvant. Booster vaccinations were administered, and samples were collected as in Study 1. Saphenous blood was collected on days 0 and 35, and the mice were euthanized on 35 dpv. In both studies, serum samples were tested for the presence of EHDV2- or EHDV6-neutralizing antibodies as described in Section 2.5.

#### 2.3.2. Cattle

Nine male Holstein calves aged approximately 3-4 months (average body weight: 102.2 kg) were verified as serologically negative against EHDV by virus neutralization assay and randomized into groups of 3. Each group received 150 μg purified rVP2 of EHDV-2 or EHDV-6 mixed with Montanide ISA25 adjuvant (Seppic, France), or PBS with adjuvant (sham-vaccinated control group) in 2 mL. Injections were administered subcutaneously twice with an interval of 3 weeks (days 0 and 21). Blood was collected via the jugular vein on 0, 21, and 35 dpv, and cattle were euthanized on 35 dpv. Serum samples were tested for the presence of neutralizing antibodies for EHDV-2 and EHDV-6 as described in Section 2.5.

#### 2.3.3. White-Tailed Deer (WTD)

Six 5-month-old male WTD, captive-bred at the United States Department of Agriculture- Agricultural Research Service (USDA-ARS), National Animal Disease Center (NADC) [27], were randomized into 2 groups (*n* = 3; see Table S1) and received 150 μg purified EHDV-2 rVP2 with Montanide ISA25 adjuvant (Seppic, France), or PBS with adjuvant (sham group) in a total volume of 2 mL at NADC. Deer were verified as serologically negative against EHDV-1, -2, and -6 at the time of vaccination by virus neutralization assays. Injections were administered SC twice with an interval of 2 weeks (0 and 14 dpv). On 22 dpv, WTD were moved from NADC to a BSL-2 facility at Kansas State University and allowed to acclimatize for eight days. Animals were housed in purpose-designed deer pens to reduce stress. At 30 dpv, all animals were challenged with 10^6.74^ pfu EHDV-2 in 2 mL total volume administered via SC and intradermal injections (0.1–0.5 mL per injection) in the lateral cervical and inguinal regions. Post-challenge, animals were monitored twice daily for general health and clinical signs of EHDV infection, including decreased activity, skin erythema, hyperemia of oral and ocular mucosa, lameness, tachypnea, and edema. Qualitative EHD clinical signs were observed by two people independently. Prior to measuring rectal temperatures and collecting blood samples, animals were sedated with xylazine (0.75–3 mg/kg; AnaSed, Lloyd Laboratories, IA, USA). Reversal of sedation was performed using a slow intravenous administration of yohimbine (0.1–0.2 mg/kg; Lloyd Laboratories). Blood was collected into serum and EDTA tubes on 0, 4, 6, 8, 12, and 14 d post challenge (dpc). Anti-coagulated blood was analyzed for complete blood cell counts (CBCs), the presence of infectious virus by virus isolation, and presence of viral RNA using RT-qPCR. Automated CBCs were determined using a large animal CBC program on an ADVIA 2120 (Siemens, Malvern, PA), followed by manual differential count (Kansas State Veterinary Diagnostic Laboratory, KSVDL). Electronic platelet quantitation was returned as “invalid” for multiple samples and thus not reported. Neutralizing antibodies in sera were detected and quantitated using virus neutralizing tests (see 2.5 below), and serum total protein concentration was determined using a hand-held refractometer (TekoPlus, Kowloon, Hong Kong). On 14 dpc, animals were sedated and subsequently euthanized using an overdose of intravenous sodium pentobarbital (minimum of 85–100 mg/kg; Fatal Plus, Vortech Pharmaceuticals, Ltd., MI, USA) with intracardiac administration of additional pentobarbital as needed in accordance with the AVMA Guidelines for the Euthanasia of Animals (2013 Edition). 

#### 2.3.4. Postmortem Examination and Analysis

After humane euthanasia, postmortem gross pathology examination was performed and tissues were sampled. Tissue samples for histological analysis were fixed in 10% buffered formalin for a minimum of 10 days prior to trimming and paraffin embedding. Fresh tissues (lung, liver, spleen, and kidney) were collected and were stored at −80 °C until processed. Following standard embedding in paraffin and the preparation of Hematoxylin and Eosin (H&E) stained slides, histopathology was performed independently by two pathologists. Tissues evaluated histologically included the heart, aorta and main pulmonary artery, lung, liver, kidney, spleen, pancreas, intestinal tract, urinary bladder, skeletal muscle and bone marrow, thymus and the prescapular, tracheobronchial, thoracic, gastrohepatic, mesenteric, and renal lymph nodes. 

### 2.4. Viruses 

EHDV-2 Alberta (SV-124-Canada 1962; ABADRU’s virus collection) and EHDV-6 Indiana (12-38993; provided by SCWDS) were used for VP2 cloning and virus neutralization tests. EHDV-2 strain cc12-304, prepared from the spleen of an infected white-tailed deer from Kansas in 2012 [28], was kindly provided by Mark Ruder, SCWDS, propagated in a *Culicoides sonorensis* cell line (CuVa W8 cells), and used for the WTD challenge [29].

### 2.5. Virus Neutralization Assay

Virus neutralization assays were performed based on Office International des Epizooties (OIE-World Organization for Animal Health) and USDA protocols [18] using EHDV-2 (Alberta 1962) and EHDV-6 (Indiana 2012). In brief, sera from immunized animals (mice, cattle, and deer) were inactivated at 56 °C for 30 min, then initially diluted 10-fold in MEM with 0.5% FBS (Atlantic Biologicals, Flowery Branch, GA) and 1x antibiotic-antimycotic (Fisher Scientific, Palatine, IL), and then 2-fold 50 μL dilutions from 1/10 to up to 1/10,240 were made in 96-well culture plates in quadruplicate. Virus with 100 TCID_50_ in 50 µL was added into each well, and plates were incubated for 1 h at 37 °C. Vero cells with 2 × 10^4^ cell per well in 100 μL of MEM with 5% FBS (Atlantic Biologicals, Flowery Branch, GA) and 1x antibiotic-antimycotic (Fisher Scientific, Palatine, IL) were added and then incubated for 5 d at 37 °C with 5% CO_2_. Wells were scored for the degree of cytopathic effects (CPE). The neutralizing antibody titer was calculated and recorded as the reciprocal of the highest serum dilution capable of preventing CPE in the respective cell culture wells. 

### 2.6. Virus Detection

#### 2.6.1. Real-time Quantitative PCR 

RT-qPCR specific for the S10 gene of EHDV serotype2 [30] was modified and performed as one-step quantitative reverse transcription real time PCR (RT-qPCR) using RNA purified from whole EDTA blood or tissue homogenates. For blood, an equal volume (250 µL) of whole blood and Qiagen RLT buffer were combined and subjected to heat (70 °C) for 5 min in a Vortemp (ISC Bioexpress, Kaysville, Utah) prior to extraction. Tissue lysates were made by processing 0.4 g of minced tissue into 1.5 mL of ATL buffer with one 5 mm stainless steel bead on a tissue homogenizer (Qiagen Tissue Lyser) using 3 rounds of 30 s bursts at 30 MHz. A 40 µL volume of protease K (Qiagen) was added, and samples were vortexed and held at room temperature overnight. A 150 µL volume of clarified lysate was mixed with 150 µL of RLT buffer and heated at 70 °C for 5 min. A 250 µL aliquot of blood lysate plus 150 µL of sterile PBS (400 µL total), or 300 µL of the RLT tissue lysate, was processed using a magnetic bead processor (Biosprint, Qiagen, Hilden, Germany) using a commercially available magnetic bead total nucleic extraction kit (GeneReach Biotechnology Corp, Lexington, MA, USA) as per the manufacturer’s recommendation with modifications; isopropyl alcohol replaced ethanol in the bead binding step, the fourth wash consisted of 100% molecular grade ethanol, and nucleic acids were eluted in 70 µL of elution buffer. 

RT-qPCR was performed using a 20 µL reaction and 5 µL of purified RNA, Quanta qScript XLT 1-step RT-qPCR Toughmix, nuclease-free water, and 0.4 µm primers and 0.1 µm probe on a CFX 96 RT-qPCR machine (BioRad, Hercules, California). The EHDV serotype 2 primer and probe set was identical to that previously published in Wilson et al. 2009 [30]: forward primer GCGTTGGATATATTGGACAAAGC, reverse primer GCATACGAAGCATAAGCAACCTT, and FAM probe TCAAATCAAACGGGCGCAACTATGG. Thermocycling conditions consisted of 50 °C reverse transcriptase step for 20 min, then a 5 min hold at 95 °C followed by 45 cycles of 95 °C for 10 s and 60 °C for 1 min. Samples were tested in triplicate with mean cycle threshold (Ct) used for gene copy number determination. Negative and positive (*in vitro* transcribed RNA [IVT RNA], see below) extraction and PCR controls were utilized for standard curve generation for the EHDV S10 gene copy number determination.

For RT-qPCR, EHDV IVT RNA was generated using the MEGAscript T7 transcription kit (ThermoFisher) using a plasmid containing a PCR-generated EHDV S10-specific amplicon as well as lyophilized PCR enzyme mix (GeneReach Biotechnology Corp, USA) and T7 promoter and terminator primers (Integrated DNA Technologies, Coralville, IA, USA). The EHDV plasmid (pBluescript II SK(-), synthesized by GenScript, Piscataway, NJ, USA), contained 410 base pairs of the EHDV S10 segment of the serotype 1 strain New Jersey Epizootic hemorrhagic disease virus (GenBank AM744986.1), which aligns 100% with serotype 2 strain 439 (Genbank AM744996.1). IVT RNA was DNase treated 3 times, column purified (MEGAclear, ThermoFisher, Waltham, MA, USA), checked for contaminating DNA with paired RT-qPCR/qPCR, and quantitated with Qubit XR and the Qubit Fluorometer (Thermofisher). EHDV gene copy number was calculated using http://www.endmemo.com/bio/dnacopynum.php. Ten-fold serial dilutions of IVT stock RNA (10^9^–10^−1^ copies/reaction) were utilized to generate a six-point standard curve using nine PCR well replicates per dilution. Copy number (CN) of the S10 gene for respective samples were mathematically determined using reference standard curve methodology, mean Ct, and the slope and intercept of the S10 gene IVT RNA standard curve. Data are reported as PCR-determined copy number/mL for blood and per reaction for tissues, which equates to approximately 2.8 mg of tissue total nucleic acid. Calculated copy numbers less than 150 (equivalent to Ct greater than 36) were considered beyond the limits of detection for this assay. 

#### 2.6.2. Virus Plaque Assay

Virus challenge material and WTD whole blood were titrated by plaque assay on Vero MARU cells (RRID:CVCL_EJ70). Confluent cell monolayers in 6-well tissue culture plates were inoculated with ten-fold serially diluted samples in 199E medium (Sigma-Aldrich, St. Louis, MO, USA), supplemented with 2% FBS and 1x penicillin/streptomycin/fungizone (PSF) (Atlanta Biologicals, Flowery Branch, GA, USA) and incubated for 1 h at 37 °C, 5% CO_2_ with rocking every 15 min. Following adsorption, the inocula were removed, and 3 mL of a 1:1 mixture of 2% carboxymethyl cellulose (Sigma-Aldrich) in 2x 199E medium (5% FBS, 2x PSF) was added. Plates were incubated at 37 °C, 5% CO_2_ for 10-14 days. Cells were fixed and stained with crystal violet fixative (25% formaldehyde, 10% ethanol, 5% acetic acid, 1% crystal violet). Virus titers were calculated from visible plaques.

### 2.7. Statistical Analyses

Statistical significance of differences between two groups was determined using a two-tailed Student’s t-test in Prism 7 (GraphPad Software, San Diego, CA); *p* < 0.05 was considered significant. 

## 3. Results

### 3.1. Neutralizing Antibody Responses in Vaccinated Mice 

To first investigate the ability of the recombinant VP2 proteins to induce neutralizing immune responses *in vivo*, mice were immunized with either EHDV-2 rVP2, EHDV-6 rVP2, an inactivated homologous virus, or adjuvant control (*n* = 5/group) at days 0 and 21. Terminal sera at 35 dpv were tested for neutralizing antibodies (Table 1). By 35 dpv, mice immunized with EHDV-2 rVP2 developed antibodies capable of neutralizing homologous virus with titers between 1:40 and 1:80. Mice immunized with EHDV-6 rVP2 had titers between 1:800 and 1:3200 against EHDV-6. No neutralizing antibodies were detected in animals vaccinated with an adjuvant control, inactivated EHDV-2, or inactivated EHDV-6.

### 3.2. Neutralizing Antibody Responses in Vaccinated Cattle 

The recombinant EHDV-2 and EHDV-6 VP2 proteins were next tested for immunogenicity in cattle. Calves were immunized with EHDV-2 rVP2, EHDV-6 rVP2, or adjuvant control on days 0 and 21 (*n* = 3/group). Serum antibody levels were measured by a virus neutralization assay (Table 2) on 0 dpv (prior to immunization), 21 dpv (after a single immunization), and 35 dpv (study end). One out of three calves immunized with EHDV-2 rVP2 developed a neutralizing titer of 1:30 by 21 dpv. By 35 dpv, the three EHDV-2 rVP2-vaccinated calves had EHDV-2 neutralizing antibody titers between 1:360 and 1:1120. Of the three calves vaccinated with EHDV-6 rVP2, two developed neutralizing antibody titers less than 1:20 by 21 dpv. By 35 dpv, neutralizing antibody titers in the three calves immunized with EHDV-6 rVP2 were between 1:160 and 1:320. No neutralizing antibodies were detected in animals vaccinated with the adjuvant alone. Cross-neutralization reactivity of EHDV-2 and EHDV-6 hyperimmune sera at 35 dpv was at or below the assay detection limit.

### 3.3. Neutralizing Antibody Responses in Vaccinated and EHDV-Challenged WTD 

**Figure 2:**
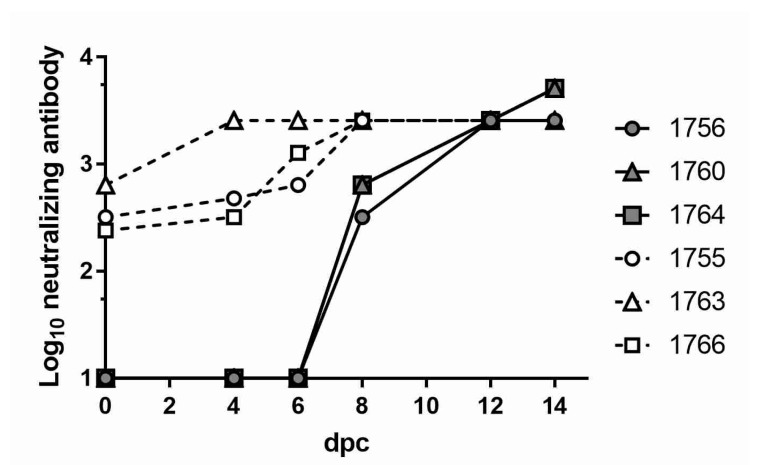
Neutralizing antibodies of white-tailed deer (WTD) challenged with EHDV-2. Sera from sham-vaccinated WTD (filled shapes, solid lines) and EHDV-2 rVP2-vaccinated WTD (open shapes, dashed lines) were collected at 0, 4, 6, 8, 12, and 14 dpc and tested for the ability to neutralize EHDV-2. Reported titers represent the reciprocal of the highest serum dilution capable of reducing cytopathic effects (CPE) more than 75%. The lowest assay dilution was 1:10; therefore, values on the x-axis (y = 1) represent titers <10. Legend indicates individual animal identification numbers.

To evaluate the efficacy of vaccine candidates in the species most susceptible to EHDV infection, the EHDV-2 rVP2 formulation, which showed a good response in cattle, was selected for a challenge study in WTD. Five-month-old captive-bred WTD were vaccinated with EHDV-2 rVP2 or sham-vaccinated with adjuvant control at 0 and 14 dpv (*n* = 3/group). At 30 dpv, all animals were challenged with 6.74 log_10_ pfu EHDV-2 (Kansas 2012). At the time of challenge (30 dpv/0 dpc), the sham-vaccinated animals had no detectable antibodies to EHDV-2 (Figure 2). Neutralizing titers of 1:10 were first detected on 6 dpc in two of the three sham-vaccinated deer and reached a maximum of 1:2560 to ≥ 1:5120 by 14 dpc. Titers in the EHDV-2 rVP2-vaccinated animals ranged from 1:240 to 1:320 prior to challenge (30 dpv/0 dpc). By 4 dpc, titers had increased to 1:320 to 1:2560, indicating an anamnestic response. Vaccinee titers reached a maximum of 1:2560 to ≥ 1:5120 by 14 dpc.

### 3.4. Clinical Parameters in EHDV-2 Challenged WTD

**Figure 3:**
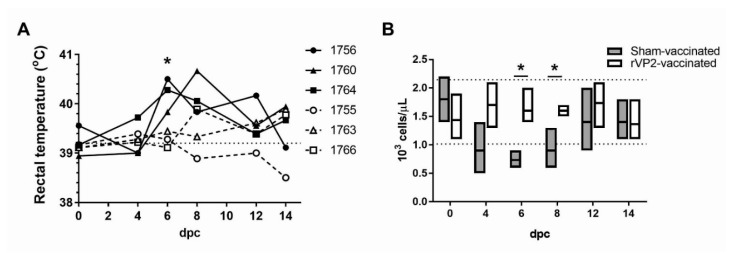
Body temperatures and peripheral lymphocyte numbers in WTD following challenge with EHDV-2. (**A**) Rectal temperatures as measured in sham-vaccinated WTD (closed shapes, solid lines) and EHDV-2 rVP2-vaccinated WTD (open shapes, dashed lines) on 0, 4, 6, 8, 12, and 14 dpc. Horizontal dotted line indicates group mean baseline temperature. Asterisk indicates *p* = 0.013. (**B**) Lymphocyte counts performed on peripheral blood samples collected from all animals at 0, 4, 6, 8, 12, and 14 dpc, excepting #1756 on 0 dpc due to the sample clotting prior to analysis. Lines on vertical bars indicate minimum, maximum, and mean absolute lymphocyte counts for the sham-vaccinated group (grey bars) and rVP2-vaccinated group (white bars) on each sample day. Horizontal dotted lines indicate the 95% confidence interval of the group baseline mean absolute lymphocyte count at 0 dpc. Asterisks indicate *p* = 0.017 (6 dpc) and *p* = 0.032 (8 dpc).

Following challenge with EHDV-2, all sham-vaccinated deer and two out of three rVP2-vaccinated deer had body temperatures exceeding the baseline mean of 39.2 + 0.20 °C on at least one day (Figure 3A). All three sham-vaccinated deer, but no rVP2-vaccinated deer, developed body temperatures exceeding 40 °C. On 6 dpc, the mean rectal temperature of the sham-vaccinated group (40.2 ± 0.20 °C) was significantly higher than the mean rectal temperature of vaccinated deer (39.3 + 0.10 °C). Beginning at 6 to 8 dpc, facial edema and increased recumbency were observed with all three sham-vaccinated animals. The most severe clinical signs were observed in animal #1764, which also developed hyperemia of the oral and ocular mucosa. EHD-related clinical signs were not observed in the WTD vaccinated with rVP2 during the study. 

Peripheral lymphocyte counts were significantly lower in the sham-vaccinated deer compared to the rVP2-vaccinated deer on 6 dpc (Figure 3B; 733 + 153 cells/µL, sham; 1600 + 346 cells/ µL, rVP2) and 8 dpc (900 + 360 cells/µL, sham; 1600 + 100 cells/µL, rVP2). Severe lymphopenia, characterized by absolute lymphocyte counts less than 1000 cells/µL, has previously been associated with severe EHD and death in WTD challenged with EHDV-2 [31]. All sham-vaccinated animals exhibited lymphocyte counts below 1000 cells/µL on at least one day, and in one animal (#1764), on four sampling days (4-12 dpi). Lymphocyte counts did not drop below 1000 cells/µL in any of the rVP2-vaccinated animals on any day. No changes were detected in the serum or plasma protein levels of the rVP2-vaccinated and sham-vaccinated animals (data not shown).

### 3.5. Blood Viral RNA Loads and Viremia in Vaccinated/Challenged WTD 

**Figure 4:**
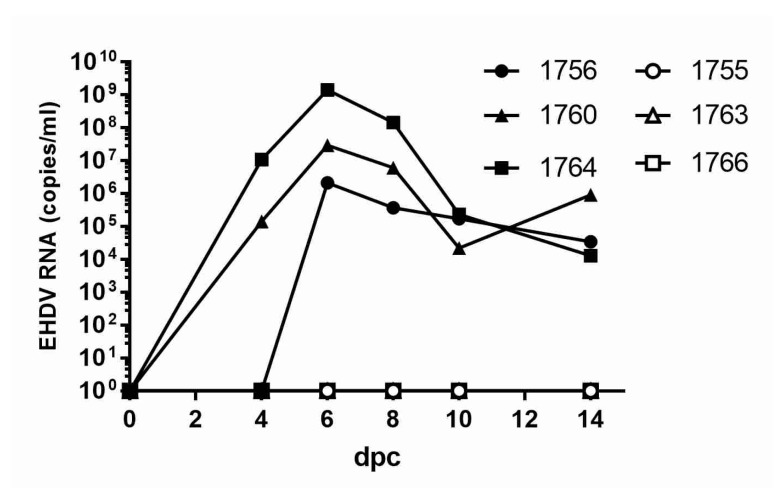
EHDV-2 RNA detected in peripheral blood of rVP2-vaccinated WTD challenged with EHDV-2. EDTA whole blood was collected from animals at 0, 4, 6, 8, 12, and 14 dpc, and EHDV RNA was quantified using the standard curve method and specific RT-qPCR for the detection of the S10 gene of EHDV-2 [30]. Closed shapes, sham-vaccinated WTD; open shapes, rVP2-vaccinated WTD.

Using RT-qPCR, EHDV-specific RNA was detected in the peripheral blood of all sham-vaccinated animals following challenge, with the peak RNAemia levels ranging from 6.33 to 9.15 log_10_ genome copies/mL on 6 dpc (Figure 4). Viral RNA was not detected in the blood of rVP2-vaccinated animals at any time point post infection. EHDV was isolated from the blood of one sham-vaccinated animal, #1764, which also had the highest levels of viral RNA in whole blood. Blood virus titers in this animal were 4.0 log_10_ pfu/mL on day 6 and 3.85 log_10_ pfu/mL on day 8, as measured by plaque assay.

### 3.6. Postmortem Analyses and Detection of Viral RNA in Tissues

**Figure 5:**
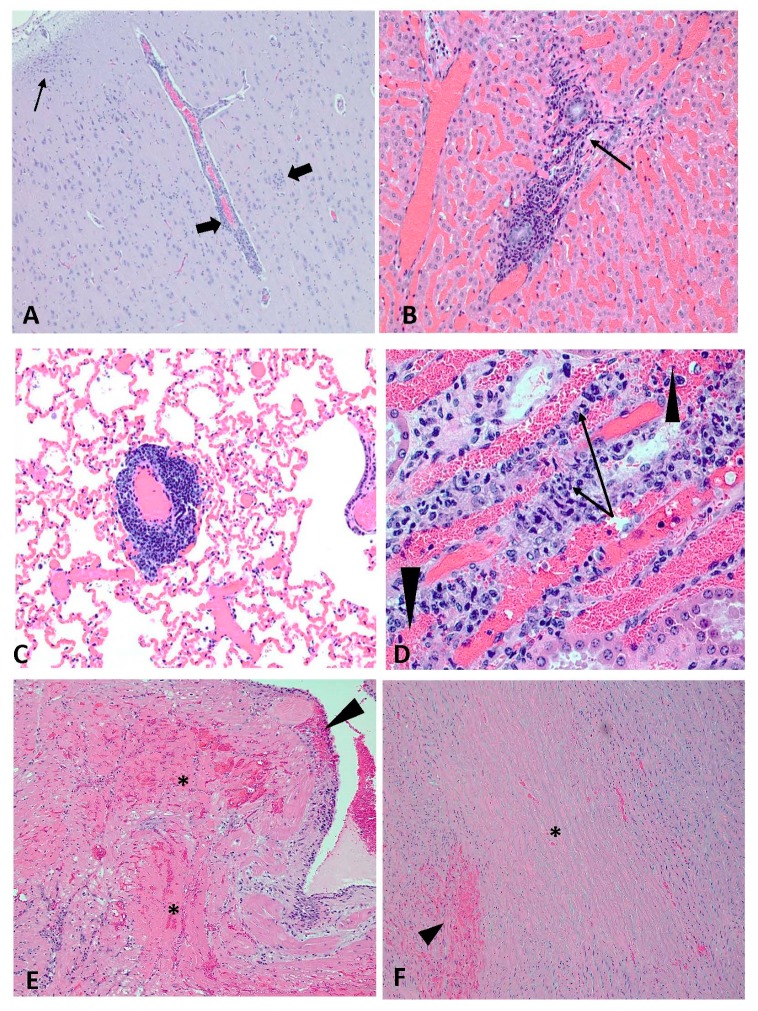
Histological lesions consistent with EHDV in sham-vaccinated WTD. (**A**) #1756; cerebral cortex; lymphoplasmacytic encephalitis with vasculitis and perivasculitis of the small vessels and large vessels (arrows) accompanied by gliosis (small thin arrow) (2X). (**B**) #1760; liver; lymphoplasmacytic vasculitis (arrow on vessel lumen) and portal/periportal hepatitis (20X). (**C**) #1756; lung; lymphoplasmacytic vasculitis and perivasculitis, medium to large veins (5X) (**D**) #1760; kidney; lymphoplasmacytic vasculitis (arrows) with hemorrhage (arrowheads) (40X). (**E**) #1764; heart; extensive multifocal myocardial necrosis and hemorrhage (*) in the papillary muscle with hemorrhage and edema of the endocardium (arrowhead) (5X). (**F**) #1764; aorta; centralized necrosis of the tunica media with loss of cellular detail (*) bordered by multifocal hemorrhage (arrowhead) (5X).

At necropsy on 14 dpc, all WTD exhibited variable pulmonary congestion and edema. Because EHDV infection, as well as handling stress, euthanasia drugs, and cardiac arrest can cause pulmonary congestion and edema, it was not possible to determine the relevance of these lesions in this study. Otherwise, no rVP2-vaccinated WTD exhibited gross and histopathological lesions attributed to EHDV infection, and no viral RNA was detected in the tissues collected from rVP2-vaccinated deer (Table 3). Gross and histopathological lesions in the three sham-vaccinated animals (#1756, #1760, #1764) were consistent with EHDV infection and characterized by necrotizing vasculitis and hemorrhagic lesions in multiple organs (Figure 5). Viral RNA was detected in two or more tissues of all sham-vaccinated, EHDV-infected deer. WTD #1764, which had the highest blood viremia as measured by RT-qPCR and plaque assay, also had the highest amount of viral RNA detected in various tissues collected at necropsy.

## 4. Discussion

Recombinant protein vaccines have inherent advantages amongst potential EHDV vaccine strategies. Unlike live-attenuated EHDV vaccines, which have the potential to reassort their gene segments with those of wild-type (field) strains [32], subunit vaccines have minimal risk of environmental impact. Because they are comprised of a limited number of selected antigens, they can also be purpose-designed with DIVA compatibility in mind, unlike live-attenuated or most inactivated virus vaccines. Specifically, immunization of animals with VP2 alone should not induce antibodies to the EHDV core protein VP7; the highly immunogenic EHDV VP7 protein is the basis of currently available EHD group-specific serological diagnostic tests for the diagnosis of EHDV infection in wild and domestic animals [33,34]. The evaluation of DIVA compatibility of the VP2 subunit vaccine is beyond the scope of this vaccine efficacy study and will be investigated in subsequent studies. 

Historically, recombinant subunit vaccine candidates have demonstrated efficacy in target host species for similar viruses such as BTV and AHSV [25,35]. Virus-like particles comprised of multiple EHDV capsid proteins have demonstrated the ability to induce neutralizing antibodies in rabbits [36]. Here, we provide evidence that a subunit formulation using recombinant EHDV serotype 2 VP2 is sufficient to protect WTD from experimental EHDV challenge with a homologous serotype virus. 

The VP2 protein of EHDV, like that of BTV, is the outermost capsid protein and contains the primary antigenic determinants for host-neutralizing antibodies [37,38]. In this study, we confirmed that a formulation using baculovirus-expressed rVP2 can induce neutralizing antibody responses *in vivo* in three different animal species and was protective in WTD after virulent EHDV challenge. The fact that neutralizing antibodies were not detected in mice vaccinated with inactivated EHDV-2 or inactivated EHDV-6 most likely was due to the virus dose used for vaccination not being high enough for inducing detectable neutralizing antibodies.

The duration of protective immunity by EHDV-2 rVP2 is not yet known and requires future investigation, which should include determination of optimal vaccination intervals and formulation for potential use in the field. Additionally, dose response studies would be useful to optimize relative potency of the rVP2 antigens. The cattle studies described herein indicate that the neutralizing antibodies generated by rVP2 proteins may be serotype specific (i.e., no significant cross-neutralization reactivity between EHDV serotypes was observed). This is not unexpected, as VP2 is the primary determinant of serotype specificity. However, with three EHDV serotypes circulating in North America and shifting epidemiological dynamics, protection from multiple serotypes would be desirable. Having demonstrated that selected rVP2 proteins are immunogenic in a monovalent vaccine formulation, efforts are currently underway to test bi- and multivalent vaccine formulations that contain rVP2 proteins from two or more serotypes to address the need for broader protection. Detailed determination of the VP2-neutralizing epitopes would also facilitate development of multi-epitope formulations. 

The clinical presentation of EHD is often highly variable in both natural and experimental infections. Some infections are subclinical, while others are chronic or insidious, and others are acutely lethal. In this study, the control sham-vaccinated EHDV-infected deer presented with signs ranging from moderate to severe, with one animal (#1764) exhibiting clear clinical and pathologic evidence of classic fulminant EHD. The variability observed in body temperatures during the study was not unexpected for a non-domesticated species; some temperature elevations observed during the study likely were attributable to the increase in activity during deer handling prior to measuring temperatures. The gross, histological, and clinical evidence of EHD in the sham-vaccinated group was not observed in the VP2-vaccinated group, both challenged with EHDV. Although all WTD exhibited variable pulmonary congestion and edema, its presence in both animal groups suggests this finding likely could be attributed to handling and/or barbiturate euthanasia. The absence of detectable viral RNA in the peripheral blood or tissues of the rVP2 vaccinees demonstrated that these animals were protected from infection following EHDV challenge. The prevention of viremia is not only relevant for protection from clinical disease, but it also addresses the need to interrupt midge transmission to naïve hosts, as deer with lower titers of viremia are less infectious to *Culicoides* vectors than are animals with high titers [5,39].

## 5. Conclusions

An experimental vaccine using baculovirus-expressed EHDV-2 rVP2 effectively induced neutralizing antibodies in two EHDV target species and mice, and provided sterilizing immunity following challenge with a homologous virulent virus in the primary target host species, WTD. Mice and cattle vaccinated with baculovirus-expressed EHDV-6 rVP2 produced homologous virus-neutralizing antibodies. The presented results indicate that a baculovirus-based EHDV VP2 vaccine could be an effective tool in preventing clinical EHD and reducing virus transmission via arthropod vectors by reducing viremia in WTD. A recombinant subunit protein-based vaccine such as this would also offer the potential for DIVA compatibility and wider safety margins than live-attenuated vaccines. The VP2 protein subunit approach investigated here, therefore, represents a promising strategy to fill an unmet need for a safe and efficacious countermeasure for EHDV.

## Figures and Tables

**Table 1:** Neutralizing antibody responses in mice vaccinated with EHDV rVP2 proteins.

**Study 1**	**Sera Collection**	**Vaccine Group**		
		Adjuvant Control	Inactivated EHDV-2	EHDV-2 rVP2
	0 dpv	-	-	-
	35 dpv	-	-	53.33 ± 19.3
**Study 2**		**Vaccine Group**		
		Adjuvant Control	Inactivated EHDV-6	EHDV-6 rVP2
	0 dpv	-	-	-
	35 dpv	-	-	1800 ± 1006

Sera from vaccinated mice were collected at 35 days post vaccination (dpv), diluted 1:10 to 1:3200, and tested for the ability to neutralize EHDV-2 (Study 1, top) or EHDV-6 (Study 2, bottom) in separate studies. Reported titers represent the reciprocal of the highest serum dilution capable of reducing cytopathic effects (CPE) more than 75%. “-” indicates neutralization was not observed at the lowest dilution; groups contain *n* = 5 mice except EHDV-6 rVP2, which contained *n* = 4 following the loss of one mouse prior to 35 dpv.

**Table 2:** Neutralizing antibody responses in cattle vaccinated with EHDV rVP2 proteins.

**Vaccine Group**	**α-EHDV-2 Titers**			**α-EHDV-6 Titers**		
	0 dpv	21 dpv	35 dpv	0 dpv	21 dpv	35 dpv
**Adjuvant Control**	-	-	-	-	-	-
**EHDV-2 rVP2**	-	10 ± 16.0	706.7 ± 384	-	-	5.8 ± 5.2
**EHDV-6 rVP2**	-	-	4.2 ± 7.0	-	5.8 ± 8.0	226.7 ± 83

The reported titers represent the reciprocal of the mean highest serum dilution capable of reducing CPE more than 75%; *n* = 3 for each group; “-” indicates neutralization was not observed at the lowest serum dilution.

**Table 3:** EHDV-2 RT-qPCR in various white-tailed deer tissues.

Tissue	Sham-Vaccinated WTD			rVP2-Vaccinated WTD		
	#1756	#1760	#1764	#1755	#1763	#1766
Lung	3.37	2.72	5.75	ND	ND	ND
Spleen	ND	2.63	3.78	ND	ND	ND
Liver	ND	3.18 (S)	3.19	ND	ND	ND
Kidney	2.53	3.43	ND	ND	ND	ND

Tissues were collected at necropsy on 14 dpc, and EHDV-2 RNA was quantified using the standard curve method and specific RT-qPCR for the detection of the S10 gene of EHDV -2. Values are reported as log10 copies of EHDV-2 RNA/reaction; “ND” = not detected; “(S)” = suspect: sample was positive in only one of 3 replicates.

## Supplementary Materials

The following is available online at https://www.mdpi.com/2076-393X/8/1/59/s1, Table S1: WTD used for the vaccine efficacy study.
