# Supplementary material for: Evaluation of A Baculovirus-Expressed VP2 Subunit Vaccine for the Protection of White-Tailed Deer (Odocoileus virginianus) from Epizootic Hemorrhagic Disease
Source: Vaccines (Basel). 2020 Jan 31;8(1):59. doi: 10.3390/vaccines8010059 (PMC7157196; doi:10.3390/vaccines8010059)
Supplement: Supplementary file 1 [file vaccines-08-00059-s001.pdf]

Table S1: WTD used for the vaccine efficacy study

Animal Number	Vaccine Group	Age at first vaccination (days)	Age at virus challenge (days)	Weight at day of challenge (kg)
1755	rVP2	164	194	31.7
1756	sham vaccine	162	192	35.9
1760	sham vaccine	160	190	30
1763	rVP2	158	188	26.3
1764	sham vaccine	155	185	32.3
1766	rVP2	151	181	33.4
